# Challenges and strategies for optimizing liver transplantation outcomes in morbidly obese cirrhotic patients: a narrative review

**DOI:** 10.1186/s12893-025-02886-w

**Published:** 2025-07-03

**Authors:** Srinivasan Muthukrishnan, Jason Zhou, Richard Wang, Jerry Bohlen, Raphael Meier, Kirti Shetty, Chandra Bhati

**Affiliations:** 1 Division of Transplantation, Department of Surgery, University of Maryland School of Medicine, Baltimore, MD 21201 USA; 2 University of Maryland School of Medicine, Baltimore, MD 21201 USA; 3 Division of Gastroenterology and Hepatology, Department of Medicine, University of Maryland School of Medicine, Baltimore, MD 21201 USA

**Keywords:** Morbidly obese, Liver transplant, Perioperative risks, Waitlist mortality, Preoperative weight loss, Bariatric surgery, robotic-assisted liver transplantation

## Abstract

The rising prevalence of morbid obesity (BMI > = 40) has introduced significant challenges in liver transplantation, particularly as metabolic dysfunction-associated steatohepatitis (MASH) becomes a leading cause of end-stage liver disease. Morbidly obese transplant candidates face higher perioperative complications, increased waitlist mortality, and worse post-transplant outcomes. This review explores these challenges and examines current strategies for managing morbid obesity in this population. Pre-operative weight loss and bariatric surgery prior to or in conjunction with transplantation have shown potential to improve eligibility and outcomes. Furthermore, emerging evidence suggests that BMI matching seems to influence graft function and survival. Finally, surgical techniques, such as robotic-assisted liver transplantation, while still novel and anecdotal, offer significant promise for reducing complications in this high-risk population.

## Introduction

The rising prevalence of obesity, and specifically morbid obesity, defined as a body mass index (BMI) > = 40, is complicating the management of patients of chronic liver disease (CLD). In the most recent survey done by the CDC, in 2023, all US states and territories had an obesity prevalence higher than 20%, with three states (Arkansas, Mississippi, and West Virginia) having a prevalence of 40% or greater [1]. The prevalence of severe obesity was 9.4% overall, with there being higher severe obesity rates in adults aged 20–39 (9.5%) and 40–59 (12.0%) compared to those aged 60 and older (6.6%) [2]. With the worsening obesity epidemic, more patients with morbid obesity are developing liver-related pathology such as metabolic dysfunction-associated steatotic liver disease (MASLD) and its inflammatory counterpart, metabolic dysfunction-associated steatohepatitis (MASH). In a couple recent studies by Ferretti et al., Wong et al. and Cholankeril et al., MASH, formerly known as non-alcoholic steatohepatitis (NASH), is the fastest growing etiology of end stage liver disease (ESLD), and ever since 2008 has surpassed alcoholic liver disease to become second leading cause of liver transplant worldwide in males [3–5] after cirrhosis secondary to Hepatitic C (HCV), and in females, the leading cause [6]. Compared with other indications for liver transplant, recipients with MASH were on average 5 years older, more likely to be female, and unsurprisingly, more likely to be obese (63% vs. 32%) [7].

While liver transplantation remains the only curative treatment for patients with ESLD, morbidly obese patients present many challenges during the laborious transplant process, including higher rates of metabolic, cardiovascular, and renal complications, worse post-operative outcomes, and high waitlist mortality. This review paper will discuss these challenges in detail, as well as how current strategies for weight loss (lifestyle interventions, pharmacological, and surgical) in the perioperative period could affect short and long-term outcomes in this population.

### Considerations in morbidly obese liver disease patients

Taking a closer look at the data surrounding liver disease in the morbidly obese, some studies show that up to 7–80% of bariatric surgery patients have MAFLD), while 25–55% of those patients progress to MASH [8]. Of the patients with MASH, about 20% will eventually progress to cirrhosis and end-stage liver disease (ESLD) [9]. Once progression to cirrhosis, management becomes more complex for those of higher body habitus, since patients become much more prone to co-morbidities such as coronary artery disease, diabetes, hypertension, dyslipidemia, metabolic syndrome and chronic kidney disease [10]. Additionally, as the cirrhosis decompensates, lifestyle measures become exponentially more difficult to implement, and bariatric surgery becomes riskier [11].

Despite the increasing need for liver transplant in morbidly obese patients, many are denied placement on the transplant waitlist due to concerns over perioperative risk, post-transplant outcomes, as well as difficulty in matching donors. Currently, the American Association for the Study of Liver Disease even considers BMI > = 40 as a soft contraindication for transplant for these exact reasons, and the European Association for the Study of the Liver guidelines state that a “multidisciplinary team should carefully evaluate patients with a BMI > = 35 before listing” [12]. When these patients do get listed, even after adjusting for common predictors of waitlist dropout, morbid obesity remained as one of the only predictors of waitlist removal or death (HR = 1.27), along with diabetes (HR = 1.14), likely from increased rates of acute-on-chronic liver failure or the many comorbid conditions discussed above [3]. These patients also had longer wait times for transplant and higher Model for End-Stage Liver Disease (MELD) scores on average [13, 14].

When these patients do meet the criteria for listing, they have worse outcomes. In a landmark systematic review done out of Italy including over a hundred thousand patients, researchers found that compared to obese patients with BMI < = 35, patients with BMI > = 40 had increased mortality risk at all time-periods up to 5 years (except at 3 years). Interestingly, however, patients whose BMI was between 35 and 40 did not show increased risk at any of these time frames. In addition, there was a significantly higher number of infections, reoperation, and vascular and cardiopulmonary complications in patients with BMI > = 30 compared to non-obese patients, with insufficient data to compare each obesity class. The length of hospital and/or ICU stay, however, was the same between obese and non-obese patients [13–15]. Conversely, a much smaller study done in the UK including two thousand patients, concluded that post-transplant survival up to 10 years is independent of recipient BMI; however biliary complication risk does correlate [5]. Yin et al. even found that a higher BMI was inversely associated with long-term mortality of patients with cirrhosis, supporting a “obesity paradox” phenomenon previously shown in various disease processes [16].

Looking more specifically at graft survival, a single-institution study showed that BMI > 35 and BMI < 35 groups had similar outcomes, while BMI < 18.5 patients had inferior graft and patient survival rates [5]. However, in another database-level study utilizing the Scientific Registry of Transplant Recipients (SRTR), morbidly obese recipients when compared with non-obese recipients showed significantly reduced 5-year graft survival (49% vs. 76%) [13].

Overall, morbidly obese patients are more likely to encounter obstacles during the transplant evaluation, tend to stay longer or have higher dropout on the waitlist, and are sicker at the time of transplant. Superobesity is associated with higher mortality, while obesity in general is associated with higher perioperative complications and reduced long term graft survival.

### Impact of donor characteristics in transplantation of morbidly obese cirrhotics

Donor-recipient BMI matching plays an important role in liver transplant regardless of BMI. Common scores proposed for matching in the graft allocation process are divided into those focusing on donor liver quality like the Donor Risk Index (DRI) and Eurotransplant-Donor-Risk-Index (ET-DRI), those focusing more on recipient or graft outcomes like the Preallocation score to predict Survival Out-comes Following Liver Transplant Score (P-SOFT), Delta-MELD (D-MELD), or Italian Score for Organ allocation (ISO), and those consider both like the Balance of Risk (BAR) score. Out of these scoring systems, only the P-SOFT score considers BMI an important variable in post-transplant outcomes [17]. In a study done out of Ohio State University, Beal et al. found that while there is increased mortality at 30 days for obese donor– obese recipient pairs after controlling for confounding variables, this difference no longer persists long term, and that 1 year and overall survival favor the obese recipient - obese donor pair. The lowest mortality rates were between non-obese donor– obese recipient pairs at 1 year, 5 years, and overall. They speculate that the early disadvantage in patients receiving obese donor livers could be due to their increased fat content resulting in graft dysfunction [1]. Another study done by Marsman et al. reinforces this theory, also reporting lower liver graft survival with livers containing up to 30% fat compared to livers without fatty infiltration; however, this effect disappeared long-term [18]. Graft and patient survival between BMI > 50 and BMI < 50 were similar as well [19]. Beal et al. also discovered that there was a shorter length of stay in the obese recipient - obese donor pair and obese recipient– non-obese donor pair, compared to the non-obese recipient– non-obese donor pair, in line with the “obesity paradox” discussed above. Proposed mechanisms for this involve a shift in the anti-inflammatory status of obese patients and their excess nutritional reserves, helping them tolerate the metabolic stress of critical illness [20, 21]. Obese living donors compared to non-obese living donors were more likely to be female, and much more likely to have diabetes and hypertension. Lifestyle modifications such as diet and exercise could improve hepatic steatosis in this donor candidate population, but the postoperative complications were comparable whether or not weight-loss was achieved, and graft and recipient survival rates were the same [22]. No studies were found looking into characteristics of deceased donor candidates. Also of note, although donor obesity was perceived as lower quality by recipients, there was no significant effects of this perceived quality on re-listing or death [23]. Despite the non-conclusive evidence detailed above on the importance of obesity in the transplant process, especially in comparison to age, gender, and MELD score, donor-recipient BMI matching should remain an important topic of continued investigation. High-resolution magic-angle-spinning nuclear magnetic resonance (HR-MAS-NMR) is an emerging technology that has potential in evaluating metabolic profiling and could prove helpful in the matching process [24].

Obese donor orthotopic liver transplant (OLT) may lead to worse short-term outcomes but not in the long term, while obese OLT recipients have shorter hospital stays but similar outcomes overall. However, tangentially related to obesity, donor-recipient body surface area (BSA) mismatch has been shown to affect rates of portal vein thrombosis within the first three months, as well as overall graft survival [25]. Liver waitlist candidates with the smallest BSAs also had a lower chance of receiving a deceased donor liver transplant compared to peers with larger body habitus [26], once again emphasizing the importance of BMI matching.

Lastly, compared to deceased donor liver transplantation (DDLT) in obese candidates, there is not much literature on living donor liver transplantation (LDLT) outside of case reports [27]. But for morbidly obese transplant candidates, LDLT may offer certain advantages like shorter cold ischemia time (CIT), shorter waitlist time, and more precise donor-recipient matching. On the other hand, the benefit of DDLT is that whole-liver grafts are potentially more appropriate for larger and more metabolically demanding patients.

### Preoperative weight loss measures

Due to the high risk of adverse outcomes linked to morbid obesity in liver transplantation, achieving weight loss during the preoperative period is an important goal. Options include lifestyle modifications, medical management, and bariatric surgery [11], but evidence on the effectiveness of lifestyle modifications or medical management for preoperative weight loss is currently limited. This may be in part due to the difficulties and risks in implementing these measures. Thorough nutritional assessment is imperative to minimize the risk of malnutrition, sarcopenia, inflammation, and fibrosis with dietary changes [11, 28, 29]. Exercise regimens are also limited by increased portal pressure and risk of variceal bleeding, which can be addressed with supervision and proper β-blockade therapy [11, 30]. With proper measures, nutritional intervention and supervised exercise programs have been shown to reduce body weight, hepatic venous pressure, incidence of sarcopenia [30], and metabolic dysfunction-associated steatotic liver disease (MASLD), damage, and fibrosis [31, 32].

Regarding medical management, diabetes medications play a crucial role due to the strong association between obesity, chronic liver disease, and diabetes. This connection is further intensified by the increased risk of persistent or new-onset diabetes, as well as MASLD, following immunosuppression after liver transplantation [33, 34]. Certain anti-obesity and diabetes drugs, particularly glucagon-like peptide-1 (GLP-1) receptor agonists and sodium glucose cotransporter 2 (SGLT2) inhibitors, have proven effective in managing diabetes in liver transplant recipients. A study by Zheng et al. compared outcomes among patients receiving GLP-1 receptor agonists, SGLT2 inhibitors, combination therapy, and dipeptidyl peptidase-4 (DPP-4) inhibitors. The results showed that GLP-1 receptor agonists, SGLT2 inhibitors, and combination therapy were associated with significant weight loss, with combination therapy also leading to lower HbA1c and alanine aminotransferase (ALT) levels [35]. Another study demonstrated that exenatide not only reduced fasting plasma glucose and promoted weight loss but also improved lipid profiles and MASLD-related markers in patients with type 2 diabetes, particularly those with a BMI ≥ 40 [36]. These drugs have shown to be well-tolerated [33, 37] and provide the additional benefit of reducing cardiovascular risk [30, 33]. While their use in pre-transplant weight loss has not yet been studied, they show great promise based on their positive effects in the post-transplant setting.

### Role of bariatric surgery

With or without non-surgical management, bariatric surgery has demonstrated significant efficacy in the context of liver transplantation, outperforming medical therapy alone [38–40]. Bariatric procedures performed pre-, intra-, and post-transplant have been shown to improve outcomes in morbidly obese patients. However, the optimal timing and choice of bariatric surgery remain areas of ongoing research, with each approach offering specific advantages and indications [40].

As previously discussed, lifestyle modifications and medical management of morbidly obese patients awaiting liver transplantation are difficult to implement and must be closely monitored. Therefore, it is important to consider bariatric surgery as an option for patients awaiting transplant. Compared to medical weight loss, bariatric surgery is associated with higher sustained weight loss and significantly lower risk of diabetes, hypertension, and recurrent and de novo MASLD post-liver transplant [39]. In Sharpton et al., out of 32 morbidly obese liver transplant candidates who failed a medically supervised weight loss program and underwent sleeve gastrectomy, 28 (88%) patients were deemed eligible for transplant within 6 months. No intraoperative deaths or liver-associated morbidity were observed [41]. A separate study done by García-Sesma et al. involving 8 patients with liver cirrhosis showed similarly safe and favorable outcomes following sleeve gastrectomy [42]. In patients with decompensated cirrhosis where bariatric surgery is contraindicated due to increased risk of intra-operative bleeding from perigastric varices, intragastric balloon placement has also shown potential in short-term weight loss and optimizing morbidly obese patients for liver transplantation [43]. With these benefits in mind, difficulties in performing bariatric surgery on these patients must be considered and approached with caution. The presence of an expanded steatotic liver and anatomical differences, specifically increased intra-abdominal fat tissue, can lead to longer operative time, intra-operative bleeding, and anastomotic leakage [44]. Rogers et al. also suggest that transplant recipients with gastric bypass likely need higher doses of tacrolimus, sirolimus and mycophenolate mofetil compared to a non-bypass patient [45], likely because these immunosuppressive drugs are mainly absorbed in the upper gastrointestinal tract [46]. Lastly, the possibility of malnutrition and liver failure associated with bariatric surgery in general must also be considered [45].

Several studies have shown that bariatric surgery can slow the progression or even lead to the resolution of MASH and MASLD, potentially reducing the need for liver transplants [45–48]. Beyond significant weight loss, improvements have been observed in various biochemical markers, including insulin resistance, glucose metabolism, hypertension, plasma lipid levels, and transaminases [45, 48, 49]. Changes in gastrointestinal hormone activity have also been reported [47, 48]. These findings suggest that bariatric surgery may be beneficial even when liver transplantation is not required, though larger studies are needed to clearly define its role in treating MASLD [48].

To reduce risks associated with pre-transplant bariatric surgery, simultaneous transplantation and bariatric surgery has been explored and shown to be a viable alternative with normal allograft function and effective weight loss [49–51]. Zamora-Valdes et al. compared outcomes in patients that underwent liver transplantation alone versus simultaneous transplantation and sleeve gastrectomy. They found that despite appropriate weight loss in both groups, liver transplantation alone was associated with weight regain while transplant with sleeve gastrectomy resulted in higher persistence of weight loss, as well as lower prevalence of hypertension, insulin resistance, and hepatic steatosis [52]. Though there are many instances of successful simultaneous transplantation and bariatric surgery, its safety and efficacy are still not well-established [50, 51].

Evidence surrounding the prevalence of bariatric surgery complications following liver transplantation is not definitive. Several studies suggest that post-transplant sleeve gastrectomy is associated with low morbidity, satisfactory long-term results, and no increase in allograft rejection [53–55]. Like pre- and non-transplant patients, steatosis and obesity-related outcomes have improved following bariatric surgery in obese, posttransplant recipients with recurrent and de novo MASLD [56]. However, a study by Osseis et al. observed one instance of early gastric fistula followed by death due to multi-organ failure 19 months later, as well as chronic infection on a parietal mesh within a group of six patients who underwent post-transplant sleeve gastrectomy [57]. Lin et al. followed nine morbidly obese patients with prior liver transplant who underwent sleeve gastrectomy. Complications in three different patients included mesh dehiscence after synchronous incisional hernia repair, bile leak from the liver surface, and postoperative dysphagia requiring reoperation [58]. These early complications could be attributed to the development of surgical adhesions following initial liver transplantation [51, 58]. As with simultaneous transplantation and bariatric surgery, larger studies are necessary to reconcile differences in the outcomes observed in these studies.

### Role of robotic surgery in morbidly obese patients with liver disease

Robotic liver surgery is gaining prominence in minimally invasive surgery due to its stable camera platform, elimination of tremors, enhanced dexterity, and improved ergonomics, making it particularly well-suited for complex liver procedures requiring precise tissue dissection and meticulous hemostasis [59]. Robotic surgery has been shown to be beneficial in morbidly obese kidney transplant recipients with fewer surgical site infections and similar graft and patient survival, and offers a safe alternative for morbidly obese patients who may otherwise be denied access to open kidney transplant [60]. However, robotic surgery in liver transplant has only been performed at a few centers worldwide and is currently selectively offered to patients with low MELD scores and low BMI. The existing literature primarily consists of anecdotal evidence, predominantly in the form of case studies that detail the successes of these procedures in one or a few patients. The following section will review the most recent literature and the role of laparoscopic and robotic-assisted liver transplantation in patients with liver disease.

The evolving role of laparoscopic techniques in liver transplantation is highlighted by recent innovations aimed at improving patient outcomes with minimal invasiveness. Dokmak et al. [61] introduces a hybrid approach combining pure laparoscopic total hepatectomy with graft implantation via a preexisting midline incision. This method demonstrates the feasibility and safety of laparoscopy in select cases, offering minimal invasiveness while retaining traditional techniques for safety. The surgery resulted in minimal blood loss and an uneventful recovery, indicating potential for further development.

Suh et al. [62] presents a pure laparoscopic LDLT where both hepatectomy and graft implantation were completed laparoscopically through a suprapubic incision. This fully laparoscopic technique provides advantages such as smaller incisions, reduced postoperative pain, and faster recovery, but requires specialized expertise due to its complexity and longer operation times. Lee et al. [63] shares a landmark case involving a 63-year-old patient with primary biliary cirrhosis who underwent total laparoscopic explant hepatectomy followed by robot-assisted engraftment. Despite a lengthy 12-hour surgery, successful completion and recovery from early allograft dysfunction illustrate the feasibility of combining robotic and laparoscopic techniques for select recipients.

In August 2023, the King Faisal Specialist Hospital in Riyadh performed the world’s first fully robotic recipient hepatectomy and liver graft implantation from a fully robotic living donor hepatectomy [64]. This approach enhances precision, safety, and visualization while requiring careful patient and graft selection, marking a significant step toward fully robotic liver transplantation (RLT).

Khan et al. [65] reported the first RLT in North America and globally using a whole graft from a deceased donor in a 62-year-old man with hepatitis C cirrhosis and hepatocellular carcinoma (HCC). The operation had a total console time of 8 h 30 min, with the patient experiencing an uneventful recovery without early allograft dysfunction or complications, further demonstrating the feasibility of RLT in carefully selected patients. A recent European series from Lisbon and Modena University Liver Transplant Centers reported six full robotic whole liver transplantations, showing favorable outcomes in patients with HCC and low MELD scores [66]. These cases highlight the potential benefits of robotic LDLT, including smaller incisions, precise tissue manipulation, and reduced postoperative pain, though further technical refinements are needed to enhance reproducibility. In the more unpredictable DDLT setting, the feasibility of robotic explant and implant is a more distant realization.

## Conclusion

In conclusion, the increased prevalence of obesity, specifically morbid obesity, has significantly changed the landscape of liver transplantation, with MASH now quickly becoming one of the top etiologies of liver failure and subsequent transplant. These patients face unique challenges throughout the transplant process, including higher rates of comorbid conditions, longer wait times, and increased mortality, but their short and long-term outcomes post-transplant seem similar to those of their weight-controlled peers. Donor-recipient characteristics play an important role in both LDLT and DDLT, but ultimately, the presence of donor obesity does not seem to impact graft function and recipient morbidity and mortality.

In addition, preoperative weight loss interventions, ranging from lifestyle modifications and pharmacological measures to bariatric surgery, are crucial in morbidly obese patients. While non-surgical measures have some supporting evidence, bariatric surgery peri-operatively has shown significant benefits, including sustained weight loss as well as reduction of hypertension and even MASH progression. Simultaneous liver transplant and bariatric surgery is also being explored as a feasible option for better long-term weight management and reduced metabolic complications, but more extensive studies are needed to establish safety and efficacy.

The early data on robotic LDLT are promising and RLT may offer reduced postoperative pain and quicker recovery times, ultimately expanding the eligibility for liver transplantation. Continued research in this field is essential to optimize robotic techniques for this high-risk population.

## Data Availability

No datasets were generated or analysed during the current study.

## References

[1] Beal EW, Tumin D, Conteh LF, Hanje AJ, Michaels AJ, Hayes D Jr, Black SM, Mumtaz K. Impact of recipient and donor obesity match on the outcomes of liver transplantation: all matches are not perfect. J Transpl. 2016;2016:9709430. 10.1155/2016/9709430. Epub 2016 Sep 1. PMID: 27688905; PMCID: PMC5023820. 10.1155/2016/9709430 PMC5023820 27688905

[2] Emmerich SD, Fryar CD, Stierman B, Ogden CL. Obesity and severe obesity prevalence in adults: United States, August 2021–August 2023. NCHS Data Brief, no 508. Hyattsville, MD: National Center for Health Statistics. 2024. 10.15620/cdc/159281 10.15620/cdc/159281 PMC11744423 39808758

[3] Ferretti S, Barreyro FJ. Worldwide increasing prevalence of Non-alcoholic steatohepatitis as an indication of liver transplantation: epidemiological view and implications. Curr Hepatol Rep. 2024;23:193–203. 10.1007/s11901-023-00628-1.

[4] Wong RJ, Singal AK. Trends in liver disease etiology among adults awaiting liver transplantation in the united States, 2014–2019. JAMA Netw Open. 2020;3(2):e1920294. 10.1001/jamanetworkopen.2019.20294. 32022875 10.1001/jamanetworkopen.2019.20294 PMC12124732

[5] Giorgakis E, Tedeschi M, Bonaccorsi-Riani E, Khorsandi SE, Menon K, Vilca-Melendez H, Jassem W, Srinivasan P, Prachalias A, Heaton N. The Effect of Recipient Body Mass Index and Its Extremes on Survival and Graft Vascular and Biliary Complications After Liver Transplantation: A Single Center Retrospective Study. Ann Transplant. 2017;22:611–621. 10.12659/aot.903475. PMID: 29026064. 10.12659/AOT.903475 PMC12577487 29026064

[6] Noureddin M, Vipani A, Bresee C, Todo T, Kim IK, Alkhouri N, Setiawan VW, Tran T, Ayoub WS, Lu SC, Klein AS, Sundaram V, Nissen NN. NASH leading cause of liver transplant in women: updated analysis of indications for liver transplant and ethnic and gender variances. Am J Gastroenterol. 2018;113(11):1649–59. 10.1038/s41395-018-0088-6. Epub 2018 Jun 8. PMID: 29880964; PMCID: PMC9083888. 29880964 10.1038/s41395-018-0088-6 PMC9083888

[7] Charlton MR, Burns JM, Pedersen RA, Watt KD, Heimbach JK, Dierkhising RA. Frequency and outcomes of liver transplantation for nonalcoholic steatohepatitis in the united States. Gastroenterology. 2011;141(4):1249–53. 10.1053/j.gastro.2011.06.061. Epub 2011 Jul 2. PMID: 21726509. 21726509 10.1053/j.gastro.2011.06.061

[8] Tran A, Gual P. Non-alcoholic steatohepatitis in morbidly obese patients. Clin Res Hepatol Gastroenterol. 2013;37(1):17–29. 10.1016/j.clinre.2012.07.005. Epub 2013 Jan 21. PMID: 23347840. 23347840 10.1016/j.clinre.2012.07.005

[9] Sheka AC, Adeyi O, Thompson J, Hameed B, Crawford PA, Ikramuddin S, Nonalcoholic Steatohepatitis. A Review. JAMA. 2020;323(12):1175–1183. 10.1001/jama.2020.2298. Erratum in: JAMA. 2020;323(16):1619. doi: 10.1001/jama.2020.5249. PMID: 32207804. 10.1001/jama.2020.2298 32207804

[10] Armstrong MJ, Adams LA, Canbay A, Syn WK. Extrahepatic complications of nonalcoholic fatty liver disease. Hepatology. 2014;59(3):1174-97. doi: 10.1002/hep.26717. Epub 2014 Jan 16. PMID: 24002776. 10.1002/hep.26717 24002776

[11] Kumar N, Choudhary NS. Treating morbid obesity in cirrhosis: A quest of holy Grail. World J Hepatol. 2015;7(28):2819–28. 10.4254/wjh.v7.i28.2819. PMID: 26668693; PMCID: PMC4670953. 26668693 10.4254/wjh.v7.i28.2819 PMC4670953

[12] Soma DMD, Park PD, Mihaylov YMD, Ekser PMD, Burcin MD, PhD1, Ghabril MMD, Lacerda MMD, Chalasani, Naga MD, Mangus RS MD1;, Kubal CA. MD, PhD1. Liver Transplantation in Recipients With Class III Obesity: Posttransplant Outcomes and Weight Gain. Transplantation Direct 8(2):p e1242, February 2022.| 10.1097/TXD.0000000000001242 10.1097/TXD.0000000000001242 PMC8735757 35018300

[13] Conzen KD, Vachharajani N, Collins KM, Anderson CD, Lin Y, Wellen JR, Shenoy S, Lowell JA, Doyle MB, Chapman WC. Morbid obesity in liver transplant recipients adversely affects longterm graft and patient survival in a single-institution analysis. HPB (Oxford). 2015;17(3):251–7. 10.1111/hpb.12340. Epub 2014 Oct 17. PMID: 25322849; PMCID: PMC4333787. 25322849 10.1111/hpb.12340 PMC4333787

[14] Singhal A, Wilson GC, Wima K, Quillin RC, Cuffy M, Anwar N, Kaiser TE, Paterno F, Diwan TS, Woodle ES, Abbott DE, Shah SA. Impact of recipient morbid obesity on outcomes after liver transplantation. Transpl Int. 2015;28(2):148– 55. 10.1111/tri.12483. PMID: 25363625. 10.1111/tri.12483 25363625

[15] Barone M, Viggiani MT, Losurdo G, Principi M, Leandro G, Di Leo A. Systematic review with meta-analysis: post-operative complications and mortality risk in liver transplant candidates with obesity. Aliment Pharmacol Ther. 2017;46(3):236–45. 10.1111/apt.14139. Epub 2017 May 10. PMID: 28488418. 28488418 10.1111/apt.14139

[16] Yin Y, Li Y, Shao L, Yuan S, Liu B, Lin S, Yang Y, Tang S, Meng F, Wu Y, Chen Y, Li B, Zhu Q, Qi X. Effect of body mass index on the prognosis of liver cirrhosis. Front Nutr. 2021;8:700132. 10.3389/fnut.2021.700132. PMID: 34490322; PMCID: PMC8417598. 34490322 10.3389/fnut.2021.700132 PMC8417598

[17] Accardo C, Vella I, Pagano D, di Francesco F, Li Petri S, Calamia S, Bonsignore P, Tropea A, Gruttadauria S. Donor-recipient matching in adult liver transplantation: current status and advances. Biosci Trends. 2023;17(3):203–10. 10.5582/bst.2023.01076. Epub 2023 Jun 22. PMID: 37344395. 37344395 10.5582/bst.2023.01076

[18] Marsman WA, Wiesner RH, Rodriguez L, Batts KP, Porayko MK, Hay JE, Gores GJ, Krom RA. Use of fatty donor liver is associated with diminished early patient and graft survival. Transplantation. 1996;62(9):1246-51. 10.1097/00007890-199611150-00011. PMID: 8932265. 10.1097/00007890-199611150-00011 8932265

[19] Vargas PA, Cullen JM, Argo C, Henry Z, Stotts MJ, Intagliata N, Northup P, Oberholzer J, Pelletier S, Goldaracena N. Liver transplantation with grafts from super obese donors. Transpl Direct. 2021;7(10):e770. 10.1097/TXD.0000000000001225. PMID: 34557587; PMCID: PMC8454911. 10.1097/TXD.0000000000001225 PMC8454911 34557587

[20] Langouche L, Marques MB, Ingels C, Gunst J, Derde S, Vander Perre S, D’Hoore A, Van den Berghe G. Critical illness induces alternative activation of M2 macrophages in adipose tissue. Crit Care. 2011;15(5):R245. 10.1186/cc10503. Epub 2011 Oct 21. PMID: 22018099; PMCID: PMC3334796. 22018099 10.1186/cc10503 PMC3334796

[21] Abhyankar S, Leishear K, Callaghan FM, Demner-Fushman D, McDonald CJ. Lower short- and long-term mortality associated with overweight and obesity in a large cohort study of adult intensive care unit patients. Crit Care. 2012;16(6):R235. 10.1186/cc11903. PMID: 23249446; PMCID: PMC3672624. 23249446 10.1186/cc11903 PMC3672624

[22] Yoon YI, Lee SG, Hwang S, Kim KH, Ahn CS, Moon DB, Ha TY, Song GW, Jung DH, Park GC. Safety of right liver donation after improving steatosis through weight loss in living donors: a retrospective study. Hepatol Int. 2024 Mar 15. 10.1007/s12072-024-10641-1. Epub ahead of print. PMID: 38485873. 10.1007/s12072-024-10641-1 38485873

[23] Dirchwolf M, Becchetti C, Stampf S, Haldimann C, Immer F, Beyeler F, Toso C, Dutkowski P, Candinas D, Dufour JF, Banz V, Swiss Transplant Cohort Study. The impact of perceived donor liver quality on post-transplant outcome. ANZ J Surg. 2023;93(4):918–25. 10.1111/ans.18217. Epub 2023 Jan 27. PMID: 36708059. 36708059 10.1111/ans.18217

[24] Faitot F, Besch C, Battini S, Ruhland E, Onea M, Addeo P, Woehl-Jaeglé ML, Ellero B, Bachellier P, Namer IJ. Impact of real-time metabolomics in liver transplantation: graft evaluation and donor-recipient matching. J Hepatol. 2018;68(4):699–706. Epub 2017 Dec 2. PMID: 29191459. 29191459 10.1016/j.jhep.2017.11.022

[25] Kostakis ID, Raptis DA, Davidson BR, Iype S, Nasralla D, Imber C, Sharma D, Pissanou T, Pollok JM. Donor-Recipient body surface area mismatch and the outcome of liver transplantation in the UK. Prog Transpl. 2023;33(1):61–8. Epub 2022 Dec 19. PMID: 36537056. 10.1177/15269248221145035 36537056

[26] Kling CE, Biggins SW, Bambha KM, Feld LD, Perkins JH, Reyes JD, Perkins JD. Association of body surface area with access to deceased donor liver transplant and novel allocation policies. JAMA Surg. 2023;158(6):610–6. 10.1001/jamasurg.2023.0191. PMID: 36988928; PMCID: PMC10061309. 36988928 10.1001/jamasurg.2023.0191 PMC10061309

[27] Taneja S. Gupta, Subash, Wadhawan, Manav, Goyal, Neerav, Single-Lobe Living Donor Liver Transplant in a Morbidly Obese Cirrhotic Patient Preceded by Laparoscopic Sleeve Gastrectomy, Case Reports in Transplantation, 2013, 279651, 2 pages, 2013. 10.1155/2013/279651 10.1155/2013/279651 PMC3872398 24386588

[28] Ravaioli F, De Maria N, Di Marco L, Pivetti A, Casciola R, Ceraso C, Frassanito G, Pambianco M, Pecchini M, Sicuro C, Leoni L, Di Sandro S, Magistri P, Menozzi R, Di Benedetto F, Colecchia A. From listing to recovery: A review of nutritional status assessment and management in liver transplant patients. Nutrients. 2023;15(12):2778. 10.3390/nu15122778. 37375682 10.3390/nu15122778 PMC10301963

[29] Andersen T, Gluud C, Franzmann MB, Christoffersen P. Hepatic effects of dietary weight loss in morbidly obese subjects. J Hepatol. 1991;12(2):224–9. 10.1016/0168-8278(91)90942-5. 2051001 10.1016/0168-8278(91)90942-5

[30] Alqahtan SA, Brown RS. Management and risks before, during, and after liver transplant in individuals with obesity. Gastroenterol Hepatol (N Y). 2023;19(1):20–9. PMID: 36865816; PMCID: PMC9972654. 36865816 PMC9972654

[31] Hohenester S, Christiansen S, Nagel J, Wimmer R, Artmann R, Denk G, Bischoff M, Bischoff G, Rust C. Lifestyle intervention for morbid obesity: effects on liver steatosis, inflammation, and fibrosis. American journal of physiology. Gastrointest Liver Physiol. 2018;315(3):G329–38. 10.1152/ajpgi.00044.2018. 10.1152/ajpgi.00044.2018 29878845

[32] Bischoff M, Zimny S, Feiner S, Sauter J, Sydor S, Denk G, Nagel JM, Bischoff G, Rust C, Hohenester S. Multidisciplinary lifestyle intervention is associated with improvements in liver damage and in surrogate scores of NAFLD and liver fibrosis in morbidly obese patients. Eur J Nutr. 2022;61(5):2725–35. 10.1007/s00394-022-02846-7. 35277756 10.1007/s00394-022-02846-7 PMC9279260

[33] Zahrawi F, Fathma S, Mehal WZ, Banini BA. Pharmacologic management of obesity after liver transplantation: A critical review. Annals Gastroenterol Dig Disorders. 2023;6(1):17–25. PMC10719957 38098758

[34] Peláez-Jaramillo MJ, Cárdenas-Mojica AA, Gaete PV, Mendivil CO. Post-Liver transplantation diabetes mellitus: A review of relevance and approach to treatment. diabetes therapy: research. Treat Educ Diabetes Relat Disorders. 2018;9(2):521–43. 10.1007/s13300-018-0374-8. 10.1007/s13300-018-0374-8 PMC6104273 29411291

[35] Zheng K, Azhie A, You X, Naghibzadeh M, Tan E, Naimimohasses S, Sridhar VS, Gupta S, Chen S, Dash S, Tsien C, Selzner N, Lilly L, Jaeckel E, Woo M, Singh S, Cherney D, Bhat M. Glucagon-like peptide-1 receptor agonists and sodium-glucose cotransporter-2 inhibitors for the treatment of diabetes mellitus in liver transplant recipients. Diabetes Obes Metab. 2024;26(10):4261–72. 10.1111/dom.15769. 39056216 10.1111/dom.15769

[36] Menekşe B, Batman A. Effect of exenatide on nonalcoholic steatohepatitis and Inflammation-Related indices in diabetic patients with Non-Alcoholic fatty liver disease. Metab Syndr Relat Disord. 2023;21(4):205–13. 10.1089/met.2022.0088. 36944132 10.1089/met.2022.0088

[37] Atthota S, Joyal K, Cote M, Scalzo R, Singh R, Consul N, Kilcoyne A, Bethea ED, Dageforde LA. Modern glucose-lowering drugs in liver transplant recipients: improvement in weight, glycemic control, and potentially allograft steatosis. Front Transplantation. 2023;2:1223169. 10.3389/frtra.2023.1223169. 10.3389/frtra.2023.1223169 PMC11235220 38993868

[38] Schauer PR, Kashyap SR, Wolski K, Brethauer SA, Kirwan JP, Pothier CE, Thomas S, Abood B, Nissen SE, Bhatt DL. Bariatric surgery versus intensive medical therapy in obese patients with diabetes. N Engl J Med. 2012;366(17):1567–76. 10.1056/NEJMoa1200225. Epub 2012 Mar 26. PMID: 22449319; PMCID: PMC3372918. 22449319 10.1056/NEJMoa1200225 PMC3372918

[39] Sharpton SR, Terrault NA, Tavakol MM, Posselt AM. Sleeve gastrectomy prior to liver transplantation is superior to medical weight loss in reducing posttransplant metabolic complications. Am J Transplantation: Official J Am Soc Transplantation Am Soc Transpl Surg. 2021;21(10):3324–32. 10.1111/ajt.16583. 10.1111/ajt.16583 33780129

[40] Ryan RJ, Heimbach JK, Diwan TD. Timing is everything: sleeve gastrectomy and liver transplantation. liver transplantation: official publication of the American association for the study of liver diseases. Int Liver Transplantation Soc. 2023;29(4):354–5. 10.1097/LVT.0000000000000005. 10.1097/LVT.0000000000000005 36809349

[41] Sharpton SR, Terrault NA, Posselt AM. Outcomes of sleeve gastrectomy in obese liver transplant candidates. Liver Transplantation: Official Publication Am Association Study Liver Dis Int Liver Transplantation Soc. 2019;25(4):538–44. 10.1002/lt.25406. 10.1002/lt.25406 PMC6535047 30588743

[42] García-Sesma A, Calvo J, Manrique A, Cambra F, Justo I, Caso O, Marcacuzco A, Loinaz C, Jiménez C. Morbidly obese patients awaiting liver transplantation-Sleeve gastrectomy: safety and efficacy from a liver transplant unit experience. Transpl Proc. 2019;51(1):33–7. 10.1016/j.transproceed.2018.01.060. 10.1016/j.transproceed.2018.01.060 30598229

[43] Choudhary NS, Puri R, Saraf N, Saigal S, Kumar N, Rai R, Rastogi A, Goja S, Bhangui P, Ramchandra SK, Raut V, Sud R, Soin A. Intragastric balloon as a novel modality for weight loss in patients with cirrhosis and morbid obesity awaiting liver transplantation. Indian J Gastroenterology: Official J Indian Soc Gastroenterol. 2016;35(2):113–6. 10.1007/s12664-016-0643-2. 10.1007/s12664-016-0643-2 27072554

[44] Sarno G, Schiavo L, Calabrese P, Álvarez Córdova L, Frias-Toral E, Cucalón G, Garcia-Velasquez E, Fuchs-Tarlovsky V, Pilone V. The impact of Bariatric-Surgery-Induced weight loss on patients undergoing liver transplant: A focus on metabolism, pathophysiological changes, and outcome in obese patients suffering NAFLD-Related cirrhosis. J Clin Med. 2022;11(18):5293. 10.3390/jcm11185293. 36142939 10.3390/jcm11185293 PMC9503676

[45] Esquivel CM, Garcia M, Armando L, Ortiz G, Lascano FM, Foscarini JM. Laparoscopic sleeve gastrectomy resolves NAFLD: another formal indication for bariatric surgery?? Obes Surg. 2018;28(12):4022–33. 10.1007/s11695-018-3466-7. 30121855 10.1007/s11695-018-3466-7

[46] Edwards A, Ensom MH. Pharmacokinetic effects of bariatric surgery. Ann Pharmacother. 2012;46(1):130–6. 10.1345/aph.1Q414. Epub 2011 Dec 20. PMID: 22190251. 22190251 10.1345/aph.1Q414

[47] Drai C, Chierici A, Pavone G, Benamran D, Alromayan M, Alamri A, Anty R, Liddo G, Iannelli A. Remission of nonalcoholic steatohepatitis after bariatric surgery: a single referral center cohort study. Surg Obes Relat Diseases: Official J Am Soc Bariatr Surg. 2024;20(5):482–9. 10.1016/j.soard.2023.10.015. 10.1016/j.soard.2023.10.015 38195314

[48] Laursen TL, Hagemann CA, Wei C, Kazankov K, Thomsen KL, Knop FK, Grønbæk H. Bariatric surgery in patients with non-alcoholic fatty liver disease - from pathophysiology to clinical effects. World J Hepatol. 2019;11(2):138–49. 10.4254/wjh.v11.i2.138. 30820265 10.4254/wjh.v11.i2.138 PMC6393715

[49] Nesher E, Mor E, Shlomai A, Naftaly-Cohen M, Yemini R, Yussim A, Brown M, Keidar A. Simultaneous Liver Transplantation and Sleeve Gastrectomy: Prohibitive Combination or a Necessity? Obes Surg. 2017;27(5):1387–1390. 10.1007/s11695-017-2634-5. PMID: 28281236. 10.1007/s11695-017-2634-5 28281236

[50] Gunturu NS, Castillo-Larios R, Bowers S, Edwards M, Burns J, Perry D, Elli EF. Combined sleeve gastrectomy with liver transplant in patients with obesity: a feasibility study. Obes Surg. 2022;32(11):3600–4. 10.1007/s11695-022-06289-1. 36169908 10.1007/s11695-022-06289-1

[51] Tariq N, Saharia A, Nwokedi U, Hobeika MJ, Mobley CM, Hsu D, Potter LM, Moore LW, Elaileh A, Sherman V, Ghobrial RM. Combined liver transplantation and sleeve gastrectomy: report of a brief-interval staged approach. Liver Transplantation Soc. 2023;29(4):422–30. 10.1002/lt.26560. Liver transplantation: official publication of the American Association for the Study of Liver Diseases and the International. 10.1002/lt.26560 35976078

[52] Zamora-Valdes D, Watt KD, Kellogg TA, et al. Long-term outcomes of patients undergoing simultaneous liver transplantation and sleeve gastrectomy. Hepatology. 2018;68(2):485–95. 10.1002/hep.29848. 29457842 10.1002/hep.29848

[53] Elli EF, Gonzalez-Heredia R, Sanchez-Johnsen L, Patel N, Garcia-Roca R, Oberholzer J. Sleeve gastrectomy surgery in obese patients post-organ transplantation. Surg Obes Relat Dis 2016 Mar-Apr;12(3):528–34. doi: 10.1016/j.soard.2015.11.030. Epub 2015 Dec 2. PMID: 26823089. 10.1016/j.soard.2015.11.030 26823089

[54] Abu-Abeid A, Dvir N, Levi S, Dayan D. Sleeve gastrectomy after liver transplantation: results of a 5-year follow-up. Minerva Surg. 2023;78(3):254–60. 10.23736/S2724-5691.22.09672-1. 36472585 10.23736/S2724-5691.22.09672-1

[55] Morris MC, Jung AD, Kim Y, Lee TC, Kaiser TE, Thompson JR, Bari K, Shah SA, Cohen RM, Schauer DP, Smith EP, Diwan TS. Delayed sleeve gastrectomy following liver transplantation: A 5-Year experience. Int Liver Transplantation Soc. 2019;25(11):1673–81. 10.1002/lt.25637. Liver transplantation: official publication of the American Association for the Study of Liver Diseases and the. 10.1002/lt.25637 31518478

[56] Ayloo S, Guss C, Pentakota SR, Hanna J, Molinari M. Minimally Invasive Sleeve Gastrectomy as a Surgical Treatment for Nonalcoholic Fatty Liver Disease in Liver Transplant Recipients. Transplant Proc. 2020 Jan-Feb;52(1):276–283. doi: 10.1016/j.transproceed.2019.11.014. Epub 2019 Dec 27. PMID: 31889539. 10.1016/j.transproceed.2019.11.014 31889539

[57] Osseis M, Lazzati A, Salloum C, Gavara CG, Compagnon P, Feray C, Lim C, Azoulay D. Sleeve gastrectomy after liver transplantation: feasibility and outcomes. Obes Surg. 2018;28(1):242–8. 10.1007/s11695-017-2843-y. 28776154 10.1007/s11695-017-2843-y

[58] Lin MY, Tavakol MM, Sarin A, Amirkiai SM, Rogers SJ, Carter JT, Posselt AM. Safety and feasibility of sleeve gastrectomy in morbidly obese patients following liver transplantation. Surg Endosc. 2013;27(1):81–5. 10.1007/s00464-012-2410-5. 22752278 10.1007/s00464-012-2410-5

[59] Becker F, Morgül H, Katou S, Juratli M, Hölzen JP, Pascher A, Struecker B. Robotic Liver Surgery - Current Standards and Future Perspectives. Z Gastroenterol. 2021;59(1):56–62. English. 10.1055/a-1329-3067. Epub 2021 Jan 11. PMID: 33429451. 10.1055/a-1329-3067 33429451

[60] Lee SD, Rawashdeh B, McCracken EKE, Cantrell LA, Kharwat B, Demirag A, Agarwal A, Brayman KL, Pelletier SJ, Goldaracena N, Fox E, Oberholzer J. Robot-assisted kidney transplantation is a safe alternative approach for morbidly obese patients with end-stage renal disease. Int J Med Robot. 2021;17(5):e2293. 10.1002/rcs.2293. Epub 2021 Jun 15. PMID: 34080270. 34080270 10.1002/rcs.2293

[61] Dokmak S, Cauchy F, Sepulveda A, et al. Laparoscopic liver transplantation: dream or reality? The first step with laparoscopic explant hepatectomy. Ann Surg. 2020;272:889–93. 31977512 10.1097/SLA.0000000000003751

[62] Suh KS, Hong SK, Lee S, et al. Pure laparoscopic living donor liver transplantation: Dreams come true. Am J Transpl. 2022;22:260–5. 10.1111/ajt.16782 34331746

[63] Lee KW, Choi Y, Hong SK, et al. Laparoscopic donor and recipient hepatectomy followed by robot-assisted liver graft implantation in living donor liver transplantation. Am J Transpl. 2022;22:1230–5. 10.1111/ajt.16943 34971490

[64] Broering DC, Raptis DA, Elsheikh Y. Pioneering fully robotic donor hepatectomy and robotic recipient liver graft implantation—a new horizon in liver transplantation. Int J Surg. 2024;110:1333–6. 38181111 10.1097/JS9.0000000000001031 PMC10942232

[65] Khan AS, Scherer M, Panni R et al. Total robotic liver transplant: the final frontier of minimally invasive surgery. Am J Transpl Off J Am Soc Transpl Am Soc Transpl Surg. 2024:S1600-6135(24)00240-5. 10.1016/j.ajt.2024.03.030 38556089

[66] Pinto-Marques H, Sobra M, Magistri P et al. Full robotic whole graft liver transplantation: A step into the future. Annals of surgery. Published online 2024. 10.1097/SLA.0000000000006420 10.1097/SLA.0000000000006420 38953528

